# Prevalence of pulp canal obliteration after traumatic dental injuries: a systematic review and meta-analysis

**DOI:** 10.1590/1807-3107bor-2024.vol38.0092

**Published:** 2024-09-30

**Authors:** Mariana Gouvêa Latini ABREU, Thaís de Oliveira FERNANDES, Leonardo Santos ANTUNES, Lívia Azeredo Alves ANTUNES, Lucianne Cople Maia de FARIA

**Affiliations:** (a) Universidade Federal Fluminense – UFF, School of Dentistry, Postgraduate Program in Dentistry, Niterói, RJ, Brazil.; (b) Universidade Federal Fluminense – UFF, School of Dentistry, Health Institute of Nova Friburgo, Nova Friburgo, RJ, Brazil.; (c) Universidade Federal do Rio de Janeiro – UFRJ, Department of Pediatric Dentistry and Orthodontics, Rio de Janeiro, RJ, Brazil.

**Keywords:** Tooth Injuries, Tooth Avulsion, Tooth Fractures, Pulp Canal Obliteration, Systematic Review

## Abstract

This systematic review aimed to answer the following question: What is the estimated prevalence of pulp canal obliteration in subtypes of traumatic dental injury (TDI) in deciduous and permanent teeth? The searches were conducted in PubMed, Embase, Scopus, Web of Science, LILACS, Grey Literature, and Google Scholar, and complemented by a manual search, until April 16^th^, 2023. Observational studies were selected based on population, exposure, and outcome (PEO) (P, deciduous or permanent teeth; E, TDI; O, pulp canal obliteration). Two reviewers (kappa 0.90) applied the eligibility criteria, extracted qualitative data, and assessed the methodological quality using the Newcastle-Ottawa tool. A meta-analysis was performed using MedCalc 17.2. Thirty-four articles were selected after screening. The methodological quality was moderate to high. The estimated prevalence of pulp canal obliteration was 27.6% (95%CI: 18.7–37.7) and 21.9% (95%CI:16.0–28.4), for permanent and deciduous teeth, respectively. Considering the TDI subtypes, the prevalence of pulp canal obliteration was higher in root fractures of the permanent teeth (78.6 %, 95%CI: 62.8–90.9) and lateral luxation injuries in deciduous teeth (29.4%, 95%CI:19.1–41.0). Our review of 34 articles of moderate and high methodological quality found that the prevalence of pulpal canal obliteration ranges from 21.9% to 27.6%. Pulp canal obliteration was most frequently detected following lateral luxation injuries of the deciduous teeth and root fractures of the permanent teeth (PROSPERO CRD42020179438).

## Introduction

The sequelae of traumatic dental injury (TDI) include pulpal necrosis, internal root resorption, external pathological root resorption, pulp calcification, and the loss of supporting tissues^1^. TDI, such as concussion and subluxation, are usually associated with minor symptoms, fewer sequelae, and limited treatment necessity^2^. Avulsion and intrusion are considered the most serious, typically associated with more profound sequelae and treatment needs^3^.

Pulp canal obliteration (PCO), also known as calcific metamorphosis, obliteration, or calcification, is characterized by calcification in the pulp cavities.^4^ The development of pulpal canal obliteration depends on two main factors: the injury and the patient’s age at the time of trauma.^5^ The most frequent PCO-related trauma types are intrusive luxation and subluxation. The most commonly affected ages are 1–4 years.^6,7^ PCO most frequently leads to a lack of pulpal sensibility^8^and yellowish crown discoloration.^9^ The pulpal response is an initial reaction to trauma, which can occur even in cases of minor trauma. Crown discoloration is caused by excessive deposition of dentin, which affects the light-transmitting properties of the tooth, leading to increased opacity.^10^

A general trend indicates that dental trauma affects one-third of children in with deciduous dentitions.^11^ The prevalence of PCO associated with traumatized deciduous teeth vary from 8.6% to 43.3%^12^. Likewise, dental trauma affects one-quarter of adolescents and adults at least once in their life.^11^ Of these, the prevalence of PCO associated with the traumatized permanent teeth ranges from 3.8% to 24%.^12^

The incidences of TDI complications have been systematically assessed.^13-16^ PCO commonly occurs after TDI. Available systematic reviews have compared the occurrence of TDI and the prevalence of PCO in cases of lateral luxation, luxation injuries, and avulsion in deciduous teeth^12,17-19^ and one analyzed concussion and subluxation in permanent teeth.^20^ However, there is no systematic review of PCO in all the subtypes of TDI in the deciduous and the permanent dentitions. This systematic review aimed to investigate the quality of existing studies and describe the overall prevalence of PCO. We also evaluated studies to determine the rate of PCO as related to each TDI. Furthermore, in determining the PCO, this review took into account that the factors related to the causes of TDI are complex. This is important because the frequency of PCO is not well-reported in the literature. This systematic review contributed a concrete and insightful assessment of TDI and its sequelae in the primary and permanent dentition.

## Methods

This systematic review was registered in the PROSPERO database (registry number: CRD42020179438) and written according to the PRISMA Statements.^21^

### Focused question

This systematic review was conducted to answer the following question: What is the estimated prevalence of PCO in subtypes of TDI in deciduous and permanent teeth?

### Strategy for identification and selection of studies

A broad literature search was performed up to April 16, 2023, using the following databases: PubMed, Scopus, Embase, Web of Science, and LILACS, via the Virtual Health Library. MeSH (Medical Subject Headings [www.nlm.nih.gov/mesh/meshhome.html]) and DECS terms (Health Sciences Descriptors [www.decs.bvs.br]), synonyms, and related terms. Boolean operators “AND” and “OR” were applied to combine the keywords (Table 1). A literature search was conducted using OpenGrey (http://www.opengrey.eu) and Google Scholar. When the data appeared to be insufficient or inconclusive, the conclusion was drawn from a critical analysis by an expert and/or consensus opinions of experienced researchers. The reference lists of the included articles were searched manually.

**Table 1:** Electronic database used and search strategy; April 16th, 2023.

Database	Search strategy
PubMed	**#1** (tooth injuries[MeSH Terms]) OR tooth avulsion[MeSH Terms]) OR tooth fractures[MeSH Terms]) OR tooth injuries[Title/Abstract]) OR tooth avulsion[Title/Abstract]) OR tooth fractures[Title/Abstract]) OR dental injuries[Title/Abstract]) OR traumatic dental injury[Title/Abstract]) OR dentoalveolar trauma[Title/Abstract]) OR tooth dislocation[Title/Abstract]) OR tooth luxation[Title/Abstract]) OR tooth intrusion[Title/Abstract]) OR dental intrusion[Title/Abstract]) OR tooth extrusion[Title/Abstract]) OR tooth subluxation[Title/Abstract]) OR tooth concussion[Title/Abstract]
	#2 (dental pulp calcification[MeSH Terms]) OR (dental pulp calcification[Title/Abstract])) OR (calcification[Title/Abstract])) OR (pulp canal obliteration[Title/Abstract])) OR (pulp calcification[Title/Abstract])) OR (dental pulp stone[Title/Abstract])) OR (calcific metamorphosis[Title/Abstract]
	**#1 and #2**
Scopus	**#1** (TITLE-ABS-KEY (tooth AND injuries) OR TITLE-ABS-KEY (dental AND injuries) OR TITLE-ABS-KEY (dental AND trauma) OR TITLE-ABS-KEY (dentoalveolar AND trauma) OR TITLE-ABS-KEY (tooth AND avulsion) OR TITLE-ABS-KEY (tooth AND dislocation) OR TITLE-ABS-KEY (tooth AND luxation) OR TITLE-ABS-KEY (tooth AND intrusion) OR TITLE-ABS-KEY (dental AND intrusion) OR TITLE-ABS-KEY (tooth AND extrusion) OR TITLE-ABS-KEY (tooth AND subluxation) OR TITLE-ABS-KEY (tooth AND fractures) OR TITLE-ABS-KEY (tooth AND concussion )
	#2 (TITLE-ABS-KEY (*dental* AND *pulp* AND *calcification*) OR TITLE-ABS-KEY (*calcification*) OR TITLE-ABS-KEY (*pulp* AND *canal* AND *obliteration*) OR TITLE-ABS-KEY (*pulp* AND *calcification*) OR TITLE-ABS-KEY (*dental* AND *pulp* AND *stone*) OR TITLE-ABS-KEY (*calcific* AND *metamorphosis*) )
	**#1 and #2**
Web of Science	**#1** (tooth injuries) *OR* **TOPIC:** (dental injuries) *OR* **TOPIC:** (traumatic dental injury) *OR* **TOPIC:** (dentoalveolar trauma) *OR* **TOPIC:** (tooth avulsion) *OR* **TOPIC:** (tooth dislocation) *OR* **TOPIC:** (tooth luxation) *OR* **TOPIC:** (tooth intrusion) *OR* **TOPIC:** (dental intrusion) *OR* **TOPIC:** (tooth extrusion) *OR* **TOPIC:** (tooth subluxation) *OR* **TOPIC:** (tooth fractures) *OR* **TOPIC:** (tooth concussion)
	*Indexes=SCI-EXPANDED, SSCI, A&HCI, CPCI-S, CPCI-SSH, ESCI Timespan=All years*
	#2 **TOPIC:** (dental pulp calcification) OR **TOPIC:** (calcification) OR **TOPIC**: (PULP CANAL OBLITERATION) OR **TOPIC:** (pulp calcification) OR **TOPIC:** (dental pulp stone) OR **TOPIC**: (calcific metamorphosis)
	Indexes=SCI-EXPANDED, SSCI, A&HCI, CPCI-S, CPCI-SSH, ESCI Timespan=All years
	**#1 and #2**
VHL (Lilacs)	tw:((tw:(tooth injuries OR dental injuries OR traumatic dental injury OR dentoalveolar trauma OR tooth avulsion OR tooth dislocation OR tooth luxation OR tooth intrusion OR dental intrusion OR tooth extrusion OR tooth subluxation OR tooth fractures OR tooth concussion )) AND (tw:(dental pulp calcification OR calcification OR pulp canal obliteration OR pulp calcification OR calcific metamorphosis OR pulp stone))) AND (db:(“LILACS”))
Open Grey/Google Scholar	tooth injuries OR traumatic dental injury OR dentoalveolar trauma and dental pulp calcification OR calcification OR pulp canal obliteration OR pulp calcification OR dental pulp stone OR calcific metamorphosis
Embase	**#1** ‘tooth injury’/exp OR ‘tooth avulsion’/exp OR ‘tooth fracture’/exp OR ‘traumatic dental injury’ OR ‘dentoalveolar trauma’ OR ‘tooth dislocation’ OR ‘tooth luxation’/exp OR ‘tooth intrusion’ OR ‘dental intrusion’ OR ‘tooth extrusion’ OR ‘tooth subluxation’ OR ‘tooth concussion’
	**#2** ‘dental pulp calcification’ OR ‘tooth pulp disease’/exp OR ‘calcification’ OR ‘pulp canal obliteration’ OR ‘pulp calcification’ OR ‘dental pulp stone’ OR ‘calcific metamorphosis’
	**#1 and #2**

### Eligibility criteria

The eligibility criteria were set as follows: population (P), deciduous or permanent teeth of any individual of any ethnicity and sex; exposure (E), any type of dentoalveolar trauma; and outcome (O), the prevalence of pulp canal obliteration in the investigated population. No restrictions were imposed on language or publication date. Studies on teeth with developmental anomalies or dental caries, patients with systemic alterations and intellectual disabilities, literature reviews, animal studies, guidelines, case reports, and records outside the proposed theme were excluded.

### Study selection

Initially, two independent examiners (MGLA and TOF) evaluated the abstracts and titles. A search alert was created for each database to identify new studies, based on the outlined search strategy. After the search, the citations found in each database were exported to the reference manager EndNote^®^, version X7 (Thomson Reuters, Philadelphia, USA). Articles that were indexed in more than one database were considered only once. Only studies that met the inclusion criteria were included in the meta-analysis. In case of doubts regarding eligibility, the article was included in the full-text analysis. Potentially eligible studies were read by the same independent examiners (MGLA and TOF). To evaluate the level of concordance between the two reviewers, 10% of the publications were randomly selected and had their ranking compared, yielding a kappa statistic of 0.90. This was calculated after abstract and full-text analyses to determine the level of agreement between the two reviewers. Data were extracted from the included studies and discussed among all authors to reach a consensus. If the information in the abstract was insufficient for the reviewers to decide, they would read the full article before making the final decision. Disagreements between reviewers were resolved after a consensus meeting with a third author (LSG).

### Data extraction

Data extraction and qualitative analyses of the selected studies. The data from the included studies were compiled and organized according to the author/year, sample, age, study design, follow-up period, TDI, number of PCO/TDI subtypes, number of PCO/other variables of interest, total PCO, and PCO-related outcomes.

During data selection and extraction, the authors were contacted via email up to three times to obtain missing data or clarify unclear information. If the authors were unable to provide the requested data or did not respond to the email within 40 days, the study was still included in the analysis based on the available information. Microsoft Translator (USA) was applied to articles that were published in languages other than English.

### Methodological quality assessment and the risk of bias

Quality assessment of the selected studies was performed by consensus between two authors (MGLA and TOF). If the reviewers disagreed, a third reviewer (LAAA) was consulted. The Newcastle-Ottawa Quality Assessment Scale was used to assess the quality of observational studies (cross-sectional and cohort studies).^22^For cross-sectional studies, the quality score was calculated based on three main categories: group selection (four items and a maximum of five stars), comparability of groups (one item and a maximum of two stars), and outcomes (two items and a maximum of three stars). The maximum score was ten points, which corresponded to studies that reached maximum stars in all categories.^22,23^For cohort studies, the quality score was calculated based on three categories: selection (four items and a maximum of four stars), comparability (one item and a maximum of one star), and outcome (three items and a maximum of four stars). The maximum score was nine points, which corresponds to studies that reached the maximum stars in all categories.^22,23^

For both types of studies (cross-sectional and cohort studies), when the score ranged from 0–4, to 5–6, and > 7 stars, the methodological quality was classified as low, moderate, or high, respectively.^22,23^

### Meta-analysis

Heterogeneity between studies was assessed using a random model. Analyses were performed using MedCalc 17.2 (MedCalc Software, Ostend, Belgium). The teeth were used as the analysis units. The following meta-analyses were performed: a) estimation of the prevalence of total pulp canal obliteration in deciduous and permanent teeth; b) estimation of the prevalence of pulp canal obliteration according to the type of trauma; and c) estimation of the prevalence of pulp canal obliteration according to the TDI, grouped according to dental tissue or supporting tissue.

In cases where some covariables influenced the stability of the outcome, sensitivity analysis or meta-regression was planned.^24^ If the sum of the included studies exceeded ten, funnel plots were generated to analyze the publication bias test.^24,25^

## Results

### Data search and study selection

**Figure 1:**
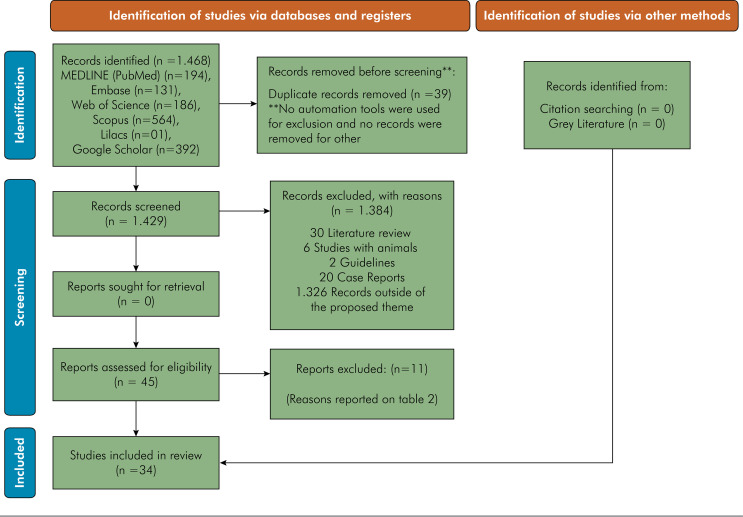
PRISMA 2020 flow diagram for new systematic reviews that included searches of databases and registers.

The study flowchart is shown in Figure 1. Initially, 1.468 studies were identified through their abstracts, which included 194,131,186, 564, 1, 0, and 392 studies from PubMed, Embase, Web of Science, Scopus, LILACS, Gray Literature, and Google Scholar, respectively. After excluding duplicate studies, 1.429 studies remained. Of these, 1.384 studies were excluded because of obvious irrelevance to the proposed theme, based on a review of the title and abstract. After reading the 45 studies in full, a second exclusion (n = 11) was performed for the reasons described in Table 2. The final selection included 34 articles.^1,2,14-16,26-55^

**Table 2:** Articles excluded after accessed in full

Reference	Reason for exclusion
Jacobsen et al., 1977	Sample selection was accessed considering the outcome (pulp canal obliteration)
Jacobsen et al., 1978	The sample was accessed considering the pulp condition, that was the outcome (pulp canal obliteration)
Andreasen et al., 1985	Repeated data. Same sample from Andreasen et al., 1989. A duplicated was removed
Andreasen et al., 1987	Repeated data. Same sample from Andeasen et al., 1989
Robertson et al., 1996	Same data from Andreasen et al., 1989
Holan et al., 2004	The sample did not encompass by the type of trauma. The patient already had to have a sequel (change in color)
Andreasen et al., 2006	Authors had excluded tooth with pulp canal obliteration
Pissiotis et al., 2007	The sample consisted of teeth that suffered trauma more than once
Lauridsen et al., 2015	Fracture of the alveolar process
Enabulele et al., 2016	The sample did not include teeth with pulp canal obliteration
Marotti et al., 2017	Fracture of the alveolar process

### Data extraction

Thirty-four studies were included in the qualitative data extraction and 34 studies were assessed (Table 3 and Table 4). Most of the studies were cross-sectional. Only one deciduous^26^ and one permanent^27^ study were cohort studies. Sixteen studies were conducted in pediatric populations and 18 in adult populations. The age of the participants ranged from 9 months^46^to 8.83 years^29^ for studies on deciduous teeth and from 5^7^ to 69^53^ years for studies on permanent teeth.

**Table 3:** Data Extraction – Permanent teeth (n=18)

Author / Year	Sample	Age	Study design	Follow-up period	Type of dental injury	Number of pulp canal obliteration/type of TDI or number of pulp canal obliteration/other variable of interest	Total pulp canal obliteration (%) (number of pulp canal obliteration/ total sample)	Outcomes related to pulp canal obliteration
Andreasen et al., 1970	Patients (n=108) Teeth (n=189)	N/R	Cross sectional	1-12 yr (mean of 3.4 yr)	Subluxation (n=78) Intrusive luxation (n=23) Extrusive Luxation (n=88)	Subluxation (20/78) Intrusive luxation (1/23) Extrusive Luxation (21/88)	22%PULP CANAL OBLITERATION (42/189)	Pulp obliteration was significantly related to the variables stage of root development, type of luxation, and crown fracture.
Jacobsen et al., 1975	Patients (N/R) Teeth (n=51)	6 - 21 yr	Cross sectional	Minimum 1 yr (mean of 6 yr)	Root fracture: -Type 1 (n=15) -Type II (n=33) -Type III (n=3)	Type I partial pulp canal obliteration (7/15) total pulp canal obliteration (1/15) Type II partial pulp canal obliteration (17/33) total pulp canal obliteration (16/33) Type III total pulp canal obliteration (3/3) Root fracture (44/51)	86%PULP CANAL OBLITERATION (44/51)	Two different patterns of pulp canal obliteration were observed: (1) partial in the apical fragments and the fracture area, and (2) progressive of the entire pulp cavity ending with almost total pulp canal obliteration. pulp necrosis did not develop as a sequel to progressive pulp canal obliteration in any case.
Rock et al., 1981	Patients (n=309) Teeth (n=517)	5 – 21 yr (mean 8.4yr)	Cross sectional	At least 2 yr	Luxation (n=133) Subluxation (n=379)	Luxation (32/133) Subluxation (49/379)	16% pulp canal obliteration (83/517)	pulp canal obliteration occurred in 24% of luxated teeth and in only 13% subluxated teeth.
Oikarinen et al., 1987	Patients (n=76) Teeth (n=172)	6 – 64 yr (mean 17.2 SD 12.3)	Cross sectional	6-32 months (mean 22 months)	Subluxation (n=48) Lateral Luxation (n=42) Extrusive Luxation (n=32) Luxation with Fracture (n=25) Avulsion (n=25)	Subluxation (12/48) Lateral Luxation (14/42) Extrusive Luxation 11/32 Luxation associated to Fracture 3/25 Avulsion 2/25	24% pulp canal obliteration (42/172)	Pulp canal obliteration of the pulp was statistically related to the age of the patient (p< 0.001) and the stage of root formation (p<0.05)
Andreasen et al., 1989	Patients (n=485) Teeth (n=637)	N/R	Cross sectional	11 years	Concussion (n=178) Extrusion (n=53) Intrusion (n=61) Lateral Luxation (n=122) Subluxation (n=223)	Concussion (n=9/178) Subluxation (n=23/223) Extrusion (n=24/53) Lateral luxation (n=34/122) Intrusion (n=3/61)	14.6 % pulp canal obliteration (93/637)	Pulp canal obliteration was more frequent among teeth with incomplete root development than among teeth in which root development was complete. pulp canal obliteration was more frequent after extrusion, lateral luxation and intrusion than after concussion and subluxation
Crona-Larsson et al., 1991	Patient (n=108) Teeth (n=171)	6 – 19yr	Cross sectional	More than 1 yr	Luxation injuries Subluxation (n=130) Extrusion (n=15) Intrusion (n=9) Lateral luxation (n=6) Exarticulated (avulsion) (n=15)	Subluxation (3/130) Lateral Luxation 2/41 Avulsion 2/11	2.9% pulp canal obliteration (5/171)	Low cases of pulp canal obliteration.
Cavalleri et al., 1995	Patient (n=55) Teeth (n=84)	6 – 12 yr	Cross sectional	Annually until 5 yr	Fracture of enamel and dentine without pulpal exposure (n=67) Fracture of enamel and dentine with pulp involvement (n=14) Fracture of enamel without pulp complications (n=3)	Fracture of enamel (0/3) Fracture of enamel and dentine without pulpal exposure (1/67) Fracture of enamel and dentine with pulp involvement (0/14)	1.5% pulp canal obliteration (without pulp involvement) (1/84)	Low cases of pulp canal obliteration (1.5%)
Çaliskan et al., 1996	Patients (n=48) Teeth (n=56)	8 – 40yr	Cross sectional	Range 2-6 yr	Root fracture (n=56)	Apical pulp obliteration (17/56) Coronal pulp obliteration (23/56) Root fracture (40/56)	75% Pulp obliteration (42/56)	High prevalence of pulp canal obliteration. Only 14 (25%) did not presented canal obliteration
Ebeleseder et al., 2000	Patient (N/R) Teeth (n=58)	Mid-term group (14.41 years) Short-term group (12.4 years)	Cross sectional	19-73 months (mean 40 months)	Intrusion (n=58) Short tem results Mid term results (n=29)	mid-term results 9/29 short-term results 6/29 Intrusion (15/58)	27% pulp canal obliteration 15/58	Teeth with pulp canal obliteration can develop pulp necrosis.
Robertson et al., 2001	Patients (N/R) Teeth (n=455)	5.3-61.3 yr	Cross sectional	6 months -17 yr	A:Uncomplicated Crown fracture without pulpal exposure/no luxation (n=106) B:Uncomplicated Crown fracture without pulp exposure associated to luxation (n=246) C:Complicated Crown fracture with pulp exposure/no luxation (n=34) D:Complicated Crown fracture with pulp exposure associated to luxation (n=69)	Crown fracture (uncomplicated or complicated without luxation (1/139) Crown fracture uncomplicated or complicated with luxation (16/315)	3.7% pulp canal obliteration (17/455)	Isolated crown fracture is rarely followed by pulp canal obliteration. crown fractures+ luxation is associated pulp canal obliteration
Lee et al., 2003	Patients (n=35) Teeth (n=55)	7.1 - 17.8 yr mean 10.6yr	Cross sectional	3, 6 months and than annually	Extrusion (n=55)	Extrusion 19/55	35% pulp canal obliteration (19/55)	The degree of extrusion (p = 0.03 0.14 (0.02-0.82) was proven to be significantly associated with the development of pulp canal obliteration
Nikoui et al., 2003	Patients (n=42) Teeth (n=58)	6.3 – 17.8yr (means11.4y)	Cross sectional	3, 6 months and than annually	Lateral Luxation (n=58)	Lateral luxation 23/58	40% pulp canal obliteration (23/58)	Extension of lateral luxation did not influenced the pulp canal obliteration occurrence (p=0.86) 0.89 (0.14-5.39)
Oginni et al., 2007	Patients (n=165) Teeth (n=168)	20-56 yr (mean age ± SD 31.3 ± 8.6 yr)	Cross sectional	N/R	Concussion (n=53) Fracture of the dental hard tissues (n=38) Subluxation (n=77)	Pulp canal obliteration (53/133) Fracture (14/38) concussion (48/53) subluxation (71/77 )	79.2% pulp canal obliteration (133/165)	Concussion and subluxation injuries resulted more in pulp canal obliteration.
Ferrazzini et al., 2008	Patients (n=26) Teeth (n=47)	7 – 59 yr	Cohort	4 yr	Lateral luxation (n=47)	Apical foramen<1 (7/47) Apical foramen ≥1 (2/47) Lateral Luxation 9/47	24.3% pulp canal obliteration (9/47)	The most frequent complication was pulp necrosis that was only seen in teeth with closed apices
Neto et al., 2009	Patient (n=12) Teeth (n=15)	7 – 14yr	Cross sectional	10-51 months (mean 26.6 months)	Intrusion (n=15)	NR Intrusion 4/15	26.7% pulp canal obliteration (4/15)	The immature teeth had six times more chances of presenting pulp canal obliteration.
Lin et al., 2016	Patients (n=166) Teeth (n=287)	6-69 yr (14.34±10.0)	Cross sectional	At least 1 yr	Uncomplicated fracture (n=47) Complicated crown fracture (n=27) Oblique fracture (n=5) Horizontal root fracture (n=8) Concussion (n=16) Subluxation (n=27) Extrusion (n=34) Lateral luxation (n=62) Intrusion (n=22) Alveolar fracture (n=6) Avulsion (n=74)	NR	4.2% pulp canal obliteration (12/287)	Pulp canal obliteration was related to time from injury to the diagnosis.
Spinas et al., 2020	Patients (n=10) Teeth (n=13)	8-16 yr	Cross sectional	5 yr	Extrusive Luxation (n=13)	Mild extrusion (n=3) Moderate extrusion (n=5) Severe extrusion (n=1)	69.23 %pulp canal obliteration 9/13	Prophylactic endodontic treatment is not appropriate for immature teeth affected by extrusive luxation injuries, given the extreme rarity of pulp necrosis in teeth already affected by pulp obliteration.
Bratteberg et al., 2020	Patients (n=338) Teeth (n=571)	16 yr	Cross sectional	NR	Moderate/Severe TDI (n=571)	NR	2.8% pulp canal obliteration 159/571	Moderate and severe TDI were more at risk of developing pulpal complications and hard tissue injuries were at higher risk of developing pulp necrosis with infection.

Footnote: yr – year; nr – not reported

**Table 4:** Data Extraction – Deciduous teeth (n=16)

Author / Year	Sample	Age (YEARS)	Study design	Follow-up period	Type of TDI	Number of pulp canal obliteration/type of TDI or number of pulp canal obliteration/ other variable of interest	Total pulp canal obliteration (%) (number of pulp canal obliteration/ total sample)	Outcomes related to pulp canal obliteration
Fried et al., 1996	Patients (n=134) Teeth (n=207)	0.8-7.5 years (mean 3.5 years)	Cross sectional	0-10 days post-trauma 11-30 days 31-91 days 92-183 days 184-365 days 366-730 days	Subluxation (n=207)	Subluxation (24/207)	24,74 % pulp canal obliteration (24/207)	Pulp canal obliteration increased in incidence and severity with time.
Boorum et al., 1998	Patient (n=287) Teeth (n=545)	3.2 years (sd 1.6) range 0.7-7.3 years).	Cross sectional	4, 8 weeks and 1 year after trauma	Concussion (n=14) Subluxation (n=140) Extrusion (n=35) Intrusion (n=91) Lateral luxation (n=186) Avulsion (n=67) No luxation (hard tissue trauma) (n=12)	Concussion (4/14) Subluxation (34/140) Extrusion (7/35) Intrusion (35/91) Lateral luxation (61/186) Avulsion (0/67) No luxation (hard tissue trauma) (0/12)	36,7% pulp canal obliteration (142/545)	The factors found to influence development of pulp canal obliteration were: displacementof the tooth at time of injury as well as detectable physiologic root resorption at time of trauma. The presence of crown fracture seemed to decrease the risk of pulp canal obliteration.
Holan et al., 1999	Patient (n=110) Teeth (n=113*) *with radiographs	12-72 months (mean 28 months)	Cross sectional	0-59 months	Intrusion (n=113)	Intrusion (58/113)	51,3% pulp canal obliteration (58/113)	Pulp canal obliteration is a common sequelae following intrusion of deciduous incisors.
Gondim & Moreira Neto, 2005	Patient (n=16) Teeth (n=22)	2 – 4 Years	Cohort	3-36 months	Intrusion (n=22)	Intrusion 0/22	0% pulp canal obliteration (0/22)	None suffered pulp canal obliteration.
Altun et al., 2009	Patient (n=78) Teeth (n=138)	12 – 48 months mean age 22.32 ± 9.72 months	Cross sectional	7 years	Intrusive injuries (n=138)	Intrusive luxation 1/41	2.5% pulp canal obliteration (1/138)	There was no relation between intrusion and pulp canal obliteration.
Mello-Moura et al., 2011	Patient (n=946) Teeth (n=1675)	0-7 years	Cross sectional	N/R	Dental tissue/dental fracture (n=551) Surrounding tissue (n=1436)	Dental tissue( dental fractute) 138/551 Support tissue 285/1436	25.3% pulp canal obliteration (423/1675)	The type of traumatic dental injury had a statistically significant effect on pulp canal obliteration. Prevalence and risk factors for pulp canal obliteration were higher among teeth suffering luxation injuries compared to those suffering injury to the dental hard tissue (p<0.05; or 1.35, 1.06<or>1.71).
Santos., 2011	Patient (n=82) Teeth (n=112)	NR	Cross sectional	15, 30, and 90 days; 5, 8, and 12 months; every 6 months until the eruption of the permanent successor	Mild TDI (crown fracture without pulp exposure, concussion, and subluxation) (n=97) Severe TDI (lateral luxation, intrusion, extrusion, and crown-tooth fracture without pulp exposure) (n=15)	Mild TDI 50/97 Severe TDI 10/15	54% pulp canal obliteration (60/112)	Occurrence of pulp canal obliteration in traumatized deciduous teeth was high. The type and recurrence of trauma were not risk factors for the development of pulp canal obliteration. There was no association between pulp canal obliteration with crown discoloration and secondary pulp necrosis
Qassen et al., 2014	Patient (n=132) Teeth (n=152)	12-70 months	Cross sectional	12-18 months	Dental fractures (including crown, crown-root fractures and root fractures, with or without pulp involvement) (n=29) Luxations (including concussion, subluxation and luxation injuries, including cases of intrusive and extrusive luxation) (n=103)	Dental Fracture 2/29 Luxations 38/103	37% pulp canal obliteration (40/152)	Considering the trauma sequelae no significant association between crown discoloration and pulp canal obliteration (p = 0.522) was detected. The association between the radicular maturity level of the traumatized teeth, and the occurrence of pulp canal obliteration (p = 0.026) after 12-18 months follow up was observed. There was an association between the type of injury and the development of pulp canal obliteration (p = 0.001) in teeth with closed apices at the time of the TDI.
Qassen et al., 2015	Patient (n=151) Teeth (n=69)	2,5 – 3,7 Years (mean)	Cross sectional	0–30 days, 31–90 days, 91–180 days, 181–365 days, 1–2 years, 2–3 years, 3–4 years, and >4 years	Intrusion (n=70) Subluxation (n=99)	Intrusion 12/70 Subluxation 20/99	18.9%pulp canal obliteration (32/169)	No significant association was evident between the type of TDI and the occurrence of sequelae (p = 0.235). Of the subluxation cases, yellow discolored teeth exhibited pulp canal obliteration (p = 0.001). In intruded teeth no association was demonstrated between pulp canal obliteration and yellow crown discoloration (p = 0.107)
Holan et al., 2016	Patients (with and without TDI) 674 Teeth with TDI 324	1,41 – 8,83 Yr	Cross sectional	N/R	Crown fracture with dentin exposure (n=53) Enamel fracture (n=256) Crown fracture with pulp exposure (n=11) Avulsion (n=4)	NR	16% pulp canal obliteration (108/674)* per patient	16% of these were pulp canal obliteration
Fontenele et al., 2017	Patient (n=55) Teeth (n=75)	12-77 months 37.6 months (±16.03)	Cross sectional	At least six-month follow-up	Concussion/Subluxation (n=23) Intrusion (n=40) Extrusion (n=6) Lateral luxation (n=8)	Concussion/Subluxation (10/23) Intrusion (6/40) Extrusion (2/6) Lateral luxation (2/8)	26.6% pulp canal obliteration (20/75)	Pulp canal obliteration was related to concussion/subluxation and extrusion (p<0.05)
Lauridsen et al., 2017a	Patient (n=205) Teeth (n=277)	1-4 yr	Cross sectional	4 weeks; 8 weeks; 6 months; 1 year after the trauma; at 6 years	Concussion (n=36) subluxation (n=241)	Subluxation (54/241) Concussion (3/36)	20.5% pulp canal obliteration (57/277)	The risk of pulp canal obliteration was significantly higher in patients aged two and four years of age.
Lauridsen et al., 2017b	Patient (n=266) Teeth (n=357)	1-4 yr	Cross sectional	4 weeks; 8 weeks; 6 months; 1 year after the trauma; at 6 years	Extrusive luxation (n=26) Lateral luxation (n=331)	Extrusion (9/26) Lateral luxation (132/331)	39.5% pulp canal obliteration (141/357)	Pulp canal obliteration occurred in all age groups. most cases (89%) were diagnosed at the 1-year follow-up examination.
Lauridsen et al., 2017c	Patient (n=149) Teeth (n=194)	1-4 yr	Cross sectional	4 weeks; 8 weeks; 6 months; 1 year after the trauma; at 6 years	Intrusion (n=194)	Intrusion (74/194)	38.1% pulp canal obliteration (74/194)	Pulp canal obliteration occurred in all age groups, but cox regression analysis showed a significantly higher risk among 3-year-old. The degree of intrusion and the presence of a concomitant crown fracture did not affect the risk of pulp canal obliteration. Half of the cases were diagnosed at the 1-year follow-up visit.
Goettems, et al., 2020	Patient (n=355) Teeth (n=628)	<2 yr 2-4 yr >4 yr	Cross sectional	7 months – 6 years	Enamel fracture (n=58) Enamel-dentin fracture (n=44) Enamel-dentin-pulp fracture (n=23) Crown-root fracture (n=21) Concussion (n=60) Subluxation (n=173) Lateral luxation (n=97) Intrusive luxation (n=113) Extrusive luxation (n=39)	NR	8.44% pulp canal obliteration (53/628)	Teeth with dark and yellow discoloration were significantly associated with pulp necrosis and pulp canal obliteration.
Sheng et al., 2021	Patient (n=45) Teeth (n=57)	NR	Cross sectional	6 months	Lateral luxation (n=57)	NR	14.04% pulp canal obliteration (8/57)	Periapical translucent image and root resorption due to periapical inflammation showed within the first three months after injury, pulp canal obliteration appeared after 6 months.

Footnote; YR – Year; NR – Not related

Table 3 reports the cases of PCO in permanent teeth. PCO was evaluated in the following TDI types: root fracture (n = 2),^4,51^ subluxation (n = 6),^14,33,34,36-38^ intrusive luxation (n = 4),^2,28,36,37^ extrusive luxation (n = 4),^34,36,40,41^ concussion (n = 2),^14,37^ lateral luxation (n = 5)^27,34,37,38,42^ and avulsion (n = 2).^34,38^

Table 4 reports the cases of PCO in deciduous teeth. PCO was evaluated in the following TDI types: root fracture (n = 1);^30^ subluxation (n = 5),^15,31,35,49,58^ intrusive luxation (n = 9),^5,15,16,29-31,35,49,58^ extrusive luxation (n = 5),^5,15,16,31,35^ concussion (n = 4),^5,15,16,31^ lateral luxation (n = 4)^5,15,47,58^ and avulsion (n = 1).^15^

### Quality assessment of individual studies

Based on the Newcastle–Ottawa methodological quality scale, the cross-sectional studies had scores ranging from four to ten points (Table 3A). Most studies (n = 23) were of high methodological quality. Of the nine articles with methodological problems, eight with minor problems were considered to have moderate methodological quality.^1,4,7,28,29,33,50,51^and only one was considered to have low methodological quality. All studies, except one^2^ did not perform sample size calculations.

Six studies did not control for confounding factors,^1,2,4,7,28,29^ and six studies had problems with the statistical tests used to analyze the data, which were not clearly described.^1,2,4,7,28,29^ According to the ascertainment of exposure (risk factor), three studies did not describe the measurement tool.^4,7,28^

Table 5 [Table t6] presents the NOS (for cohort studies) of the two prospective studies^26,27^Both had good methodological quality (eight stars), with problems in the selection section (demonstration that the outcome of interest was not present at the start of the study).

**Table 5:** Evaluation of methodological quality assessment according New Castle

Autor/year	Selection				Comparability	Outcome		Total star (0-10)	Quality Assessment
	S1	S2	S3	S4	C	O1	O2		
Andreasen et al., 1970			*	**	**	**	*	8	High
Jacobsen et al., 1975			*	**		**		5	Moderate
Rock et al., 1981			*	**		**	*	6	Moderate
Oikarinen et al., 1987			*	**	**	**	*	8	High
Andreasen et al., 1989			*	**	**	**	*	8	High
Crona Larsson et al., 1991			*	**	**	**		7	High
Cavalleri et al., 1995			*	**		**	*	6	Moderate
Çaliskan et al., 1996			*	**	*	**		6	Moderate
Fried et al., 1996			*	**	**	**	*	8	High
Boorum et al., 1998			*	**	**	**	*	8	High
Holan et al.,1999			*		**	**	*	6	Moderate
Ebeleseder et al., 2000			*			**	*	4	Low
Robertson et al., 2001			*	**		**	*	6	Moderate
Lee et al., 2003			*	**	**	**	*	8	High
Nikoui et al., 2003			*	**	**	**	*	8	High
Oginni et al., 2007			*	**	*	**	*	7	High
Altun et al., 2009			*	**	*	**	*	7	High
Neto et al., 2009			*	**	*	**		6	Moderate
Mello-Moura et al., 2011			*		*	**	*	5	Moderate
Zimmerman et al., 2011			*	**	**	**	*	8	High
Qassen et al., 2014			*	**	**	**	*	8	High
Qassen et al., 2015	*	*	*	**	**	**	*	10	High
Lin et al., 2016			*	**	**	**	*	8	High
Holan et al., 2016			*	**	*	**	*	7	High
Fontenele et al., 2017			*	**	*	**	*	7	High
Lauridsen et al., 2017a			*	**	**	**	*	8	High
Lauridsen et al., 2017b			*	**	**	**	*	8	High
Lauridsen et al., 2017c			*	**	**	**	*	8	High
Goettems et al., 2020			*	**	*	**	*	7	High
Spinas et al., 2020			*	**	**	**	*	8	High
Brattenberg et al., 2020			*	**	*	**	*	7	High
Sheng et al., 2021			*	**	**	**	*	8	High

**Table 6 t6:** Evaluation of methodological quality assessment according New Castle - Otawwa Scale – Cohort Studies

Autor/year	Selection				Comparability	Outcome			Total star (0-9)	Quality Assessment
	S1	S2	S3	S4	C	O1	O2	O3		
Gondim & Moreira Neto, 2005	*	*	*		*	**	*	*	8	High
Ferrazzini et al., 2008	*	*	*		*	**	*	*	8	High

Good quality: 3 or 4 stars in selection domain AND 1 or 2 stars in comparability domain AND 2 or 3 stars in outcome/exposure domain.

Fair quality: 2 stars in selection domain AND 1 or 2 stars in comparability domain AND 2 or 3 stars in outcome/exposure domain.

Poor quality: 0 or 1 star in selection domain OR 0 stars in comparability domain OR 0 or 1 stars in outcome/exposure domain.

### Meta-analysis

**Figure 2:**
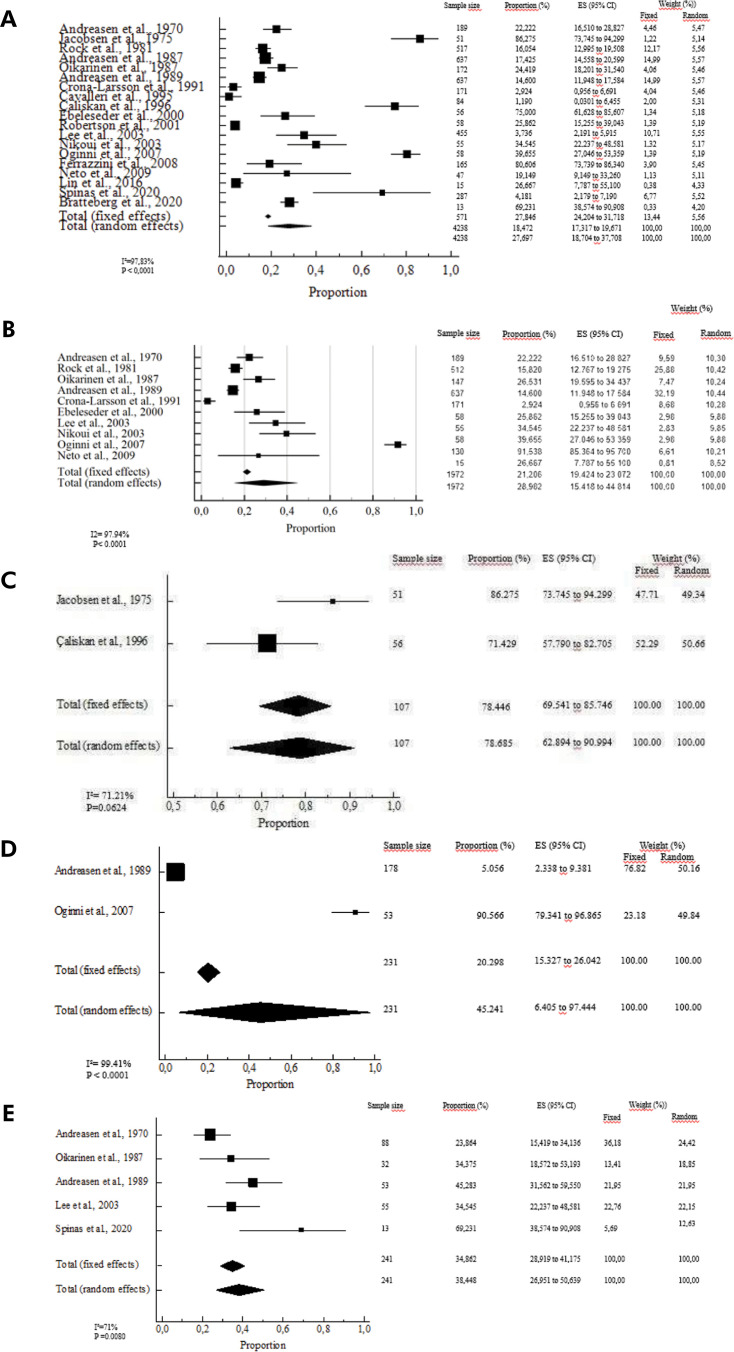
Meta-analysis evaluation showing the prevalence rates of pulp canal obliteration of all included studies in permanent teeth.

The unit of analysis in the meta-analysis was the number of teeth presented in the articles. According to the random model, the estimated prevalence of PCO in permanent teeth was 27.6% (95%CI: 18.7–37.7) (Figure 2A). This analysis showed significant heterogeneity among the studies (p < 0.005). The estimated prevalence for TDI grouped according to support tissues was 28.9% (95%CI: 15.4–44.8; p < 0.0001) (Figure 2B) and 33.0% (95%CI: 2.7–75.9; p < 0.0001), respectively. According to the random model, root fractures were most frequently associated with PCO (78.6 %, 95%CI: 62.8–90.9, p = 0.0624; Figure 2C); followed by concussion (45.2%, 95%CI: 6.4–97.4, p < 0.0001) with high heterogeneity (Figure 2D); and extrusive luxation (38.4%, 95%CI: 26.9–50.6, p = 0.0080; Figure 2E). The estimated prevalence of the other TDI subtypes are as follows: 25.7% subluxation, 24.4% lateral luxation, 14.4% intrusive luxation, 12.9% avulsion, and 8.1% crown fracture.

**Figure 3:**
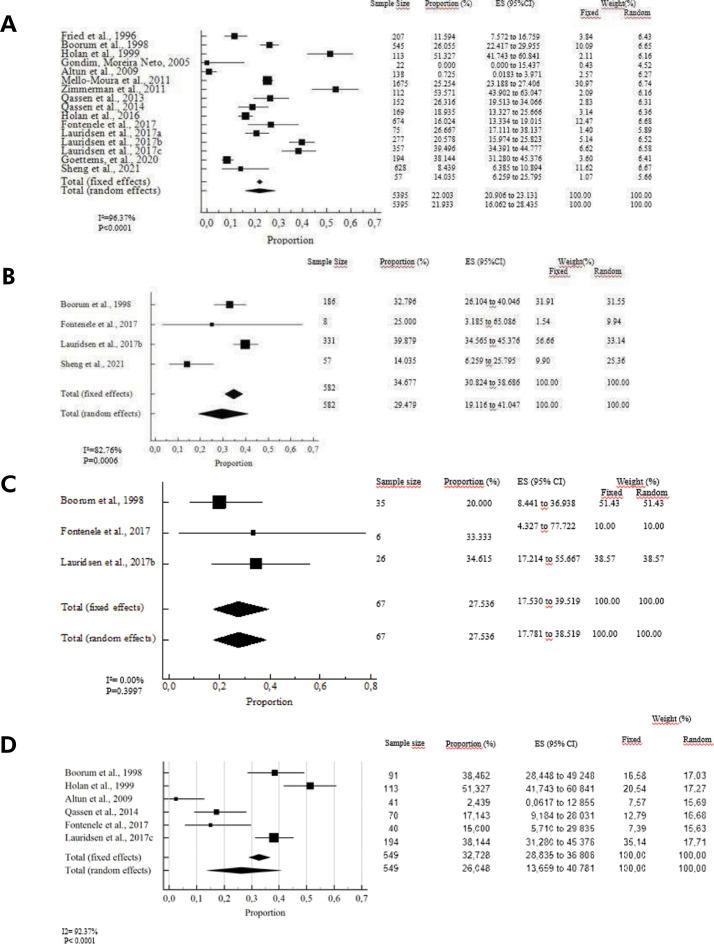
Meta-analysis evaluation showing the prevalence rates of pulp canal obliteration in root fracture in permanent teeth.

The estimated prevalence of PCO in deciduous teeth was 21.9% (95%CI: 16.0–28.4) in the random model (Figure 3A). Significant heterogeneity among the studies was noted (p < 0.005). According to the random model, PCO was more frequent in teeth affected by lateral luxation (29.4%, 95% CI: 19.1–41.0, p = 0.0006) with low heterogeneity (Figure 3B), followed by extrusive luxation (27.5%, 95% CI: 17.5 to 39.5, p = 0.3997) with low heterogeneity (Figure 3C), and intrusive luxation (26.04%, 95% CI: 13.6–40.7, p < 0.0001) with high heterogeneity (Figure 3D). The estimated prevalence of the other TDI subtypes was: 19.42% for subluxation and 17,14% for concussion.

**Figure 4:**
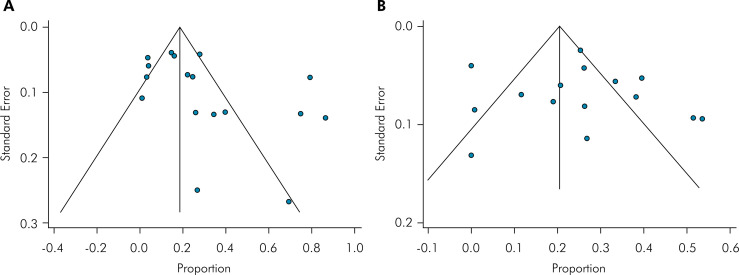
Meta-analysis evaluation showing the prevalence rates of pulp canal obliteration in all included studies in deciduous teeth

The potential risk of publication bias was evaluated through visual analysis of the funnel plots, with roughly symmetrical funnel plots indicating low risk and asymmetrical funnel plots indicating high risk. The funnel plots of the permanent and deciduous studies appeared asymmetric with outliers toward the right (Figures 4A and 4B). The stability of the outcome was not influenced by covariates. Consequently, sensitivity analysis or meta-regression was not indicated. Supplemental figures are available on https://osf.io/5hbrq/

## Discussion

The prevalence of PCO in the deciduous and permanent dentitions did not show a large difference. However, when considering all types of TDI, lateral luxation was most frequently associated with PCO in deciduous teeth, and root fractures were most frequently associated with PCO in the permanent dentition.

The prevalence of PCO in deciduous teeth was lowest in cases of concussion and subluxation. Generally, studies have reported that deciduous teeth with concussion or subluxation carry a low risk of pulp necrosis, periapical inflammation, root resorption, and premature tooth loss.^26,45^ In contrast, some studies included in this systematic review found that teeth that experienced subluxation may develop increasing sequelae (frequency and severity) over time, especially in patients, aged two and four years.^15,55^

In deciduous teeth, PCO was more commonly associated with lateral luxation, followed by extrusive, and then, intrusive luxation. One reason for this may be that luxation is more often associated with complications, such as external or replacement root resorption as a result of damage to the surrounding tissues, including the periodontal ligament.^56^ In addition, revascularization can occur even if deciduous teeth are not repositioned after luxation injuries. The teeth that are left in the luxated position are usually immobile, whereas those that are repositioned and not splinted tend to be mobile. Mobility may facilitate bacterial progression along the injured PDL, resulting in further sequelae.^57^

In permanent teeth, PCO is more prevalent in cases of root fractures, concussions, and extrusive luxation. Certain types of TDI, such as extrusive luxation^43^ and lateral luxation, are associated with a greater likelihood of PCO than pulp necrosis.^10^ Several factors influence the type of tissue repair following root fractures. These factors include the root development stage, repositioning of the dislocated fragments, and any associated signs and symptoms, such as mobility and pain. PCO is the most common sequela of root fractures in permanent teeth.^42^ Reparative dentin is deposited on the canal walls, concentrating along the fracture line, and more fibroblasts are found in this region than in the apical portion, where the pulp remains more vascularized.^49^

Thus, the high prevalence of PCO after root fractures (29.4% to 95.2%) in permanent teeth is noteworthy.^42^ This is irrespective of the location of the fracture. Our finding (78.6%) is similar to another study, which reported a PCO prevalence of 75% after root fractures in permanent teeth.^43^ We found that the lowest prevalence of PCO was observed among crown fractures (8.1%). Bacteria invading the exposed dentin is one of the most important factors leading to irreversible inflammatory pulpal changes. Conversely, inflammatory changes are transient when bacterial invasion is prevented. In teeth with intact pulpal circulation, dentin is resistant to bacterial invasion.^50^

This systematic review was conducted to answer the following question: “What is the estimated prevalence of PCO in subtypes of TDI in deciduous and permanent teeth?” After a systematic search and application of the predetermined eligibility criteria, 34 articles were selected.

To control for the probable risk of bias in this systematic review and meta-analysis, a search was performed using a considerable number of databases for all bibliographic references of the selected articles. MeSH terms and keywords were used for articles published in this area to minimize inconsistencies and the possibility of not finding potentially eligible studies. Gray literature was used to identify unpublished and ongoing studies and studies in other languages, which were analyzed independently by two reviewers (selection process, quality assessment, and data extraction).

The estimated prevalence of PCO in deciduous and permanent teeth showed significant heterogeneity among the eligible studies. Data collected using funnel plots showed asymmetry, suggesting a publication bias. These biases are most likely due to differences in the study population, study design, and follow-up duration. Consequently, our results should be interpreted with caution. Further analyses with more data are required to determine other study-related factors that may have contributed to the heterogeneity observed in this study.

The severity of the sequelae caused by TDI depends on various factors, such as the trauma type, the age of the child, and the treatment provided^51^. The age at which TDIs occur is also an important consideration when developing strategies to predict and prevent serious consequences affecting the developing permanent successors.^52^

The ages of the patients included in the studies ranged from 9 months to 8.83 years. Studies have generally reported a higher frequency of TDI at one, two, and four years of age.^53-55^ In addition, one study showed that the risk of PCO was significantly higher in patients, aged two and four years. The ages of patients who experienced PCO in their permanent teeth were highly varied (5 to 69 years).

The literature suggests that 7%–27% of teeth with PCO will develop pulp necrosis, with radiographic signs of periapical disease.^5,56,57^ However, only two articles in the permanent group reported this.^2,27^ Furthermore, this was not reported in studies of deciduous teeth. Although pulp necrosis evaluation was not the objective of this systematic review, more studies should be conducted to explain this correlation.

A Newcastle–Ottawa methodological quality assessment was used to determine whether the research methods and results were sufficiently valid. The most common problems were related to sampling issues, for which no sample calculations were reported. This may have influenced the reproducibility and interpretation of the results of these studies. Another relevant aspect is the study type. Most studies were retrospective; hence, the development of the lesion may not have been monitored, and some data may not be as reliable as those in prospective studies.

This study has some limitations. First, the use of translation software for articles written in other languages and resultant translations may have led to a loss of relevant information.

We did not consider differences by sex and we did not detect a positive association between PCO and males vs. females. This aspect should be considered in future studies.

This study found a high prevalence of PCO after TDI in deciduous and permanent teeth. In particular, we noted that PCO occurred most frequently in cases of lateral luxation in deciduous teeth and root fractures in permanent teeth. Our findings should be considered when reviewing or developing preventive strategies. Moreover, our results highlight the importance of a correct diagnosis, treatment planning, and follow-up, in determining favorable outcomes. Dental professionals dealing should be prepared to identify, treat, or refer patients for appropriate treatment, where necessary. Our findings also highlighted the need to design reliable studies to reduce imprecision and variability.

## Conclusion

Based on studies of moderate and high methodological quality, the prevalence of PCO ranges from 22% to 27.6%. Lateral luxation in deciduous teeth and root fractures in permanent teeth demonstrated the highest prevalence of PCO.
